# Punicalagin is the key pomegranate polyphenol inhibiting gut microbial trimethylamine (TMA) production from l-carnitine in an *in vitro* human colon model

**DOI:** 10.1039/d5fo04781a

**Published:** 2026-02-02

**Authors:** J. E. Haarhuis, M. I. Gamal El-Din, D. Lamprinaki, P. A. Kroon

**Affiliations:** a Quadram Institute Bioscience, Research Park Rosalind Franklin Rd Norwich NR4 7UQ UK paul.kroon@quadram.ac.uk; b Department of Pharmacognosy, Faculty of Pharmacy, Ain Shams University Abbasia Cairo 11566 Egypt

## Abstract

TMAO has been linked to various cardiometabolic diseases and all-cause mortality risk. A major dietary precursor of TMAO is l-carnitine. l-Carnitine is metabolised by microbiota to γ-butyrobetaine (γ-BB), followed by trimethylamine (TMA), and is then oxidised to TMAO in the liver. Previously, we have shown that a polyphenol-rich pomegranate extract dose-dependently inhibited the production of γ-BB and TMA from l-carnitine. Here, we further investigated the effects of the pomegranate extract and its individual constituents/metabolites (polyphenols, spray-drying agent gum Arabic, and urolithins) on the microbial metabolism of l-carnitine to γ-BB and TMA using a high-throughput *in vitro* model of the human colon. A small-scale, high-throughput colon model was inoculated with l-carnitine, individual constituents of the extract (2 mg mL^−1^), and 1% human faecal inoculum, while continuously monitoring pH. Samples were collected over 48 hours, and methylamines were quantified using LC-MS/MS with isotopically labelled internal standards. Punicalagin, but not the other constituents, inhibited the conversion of l-carnitine to γ-BB (*p* < 0.001) and almost completely blocked TMA production compared to the control (*p* < 0.003). Furthermore, including the whole pomegranate extract in the high-throughput colon model significantly reduced the pH and completely inhibited l-carnitine metabolism, suggesting that acidification may also inhibit microbial l-carnitine metabolism. Here it was shown that, of all the tested phenolic and non-phenolic components of the pomegranate extract, only punicalagin inhibited TMA production from l-carnitine, highlighting it as a promising inhibitor of TMA and potentially TMAO formation.

## Abstract 2

Punicalagin is the single pomegranate polyphenol inhibiting the production of proatherogenic trimethylamine (TMA) from l-carnitine *in vitro*. Dietary l-carnitine is converted by gut microbiota to TMA. Created in BioRender.

## Graphical abstract

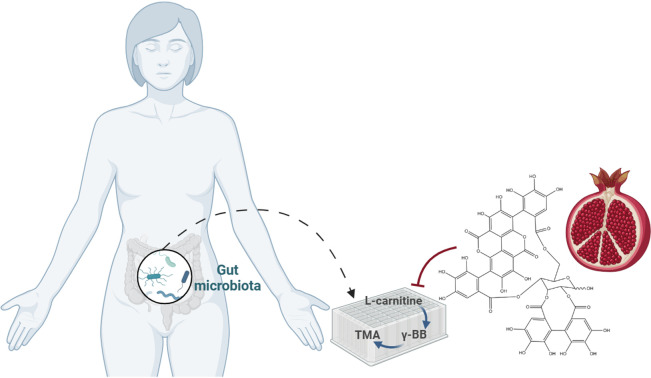

## 1 Introduction

Emerging research has suggested a connection between the gut microbiome and cardiovascular disease (CVD) development.^1^ The gut microbiome generates various metabolic compounds that may contribute to an increased cardiovascular risk, including phenylacetylglutamine,^2^ imidazole propionate,^3^ and trimethylamine *N*-oxide (TMAO).^4,5^ Among these metabolites, TMAO has received the most extensive research attention.

Epidemiological evidence demonstrated an association between TMAO levels and cardiometabolic disease and all-cause mortality risk.^6^ For instance, a large-scale prospective study examining 6767 adults across multiple ethnic groups (comprising 38% white, 28% black, 22% Hispanic, and 12% Chinese participants) revealed a dose–response relationship between plasma TMAO concentrations and atherosclerotic CVD risk.^7^ The study found that hazard ratios increased by 23% and 33% for plasma TMAO concentrations of 4.98 and 9.20 µM respectively, when compared to the lowest quartile of 1.75 µM. Further supporting this connection, an intervention study administered daily choline supplementation to healthy volunteers over two months.^8^ Within four weeks of choline treatment, participants exhibited a more than ten-fold elevation in fasting plasma TMAO levels, which remained elevated throughout the study period. Concurrently, researchers observed increased adenosine diphosphate-induced platelet aggregation that correlated significantly with rising plasma TMAO levels.

l-Carnitine is a primary precursor of TMAO. The metabolic pathway begins when gut microbiota convert l-carnitine into γ-butyrobetaine (γ-BB), which is subsequently transformed into trimethylamine (TMA). Finally, hepatic enzymes oxidise TMA to produce TMAO. Given this pathway, interventions that reduce TMA formation could potentially mitigate CVD risk by decreasing subsequent TMAO production.

Dietary polyphenols present particularly promising candidates for such interventions. The absorption of polyphenols in the small intestine is low,^9^ allowing them to reach the colon where they can directly interact with gut microbiota. Additionally, polyphenols demonstrate antibacterial,^10^ anti-inflammatory,^11,12^ and anti-oxidative^11^ properties. Our previous research demonstrated that a pomegranate extract rich in polyphenols could dose-dependently suppress both γ-BB and TMA production from l-carnitine in an *in vitro* batch colon model.^13,14^

The specific bioactive components responsible for the observed TMA-inhibiting effect of the pomegranate extract have not yet been identified. Characterisation of the extract revealed that the extract contained 5.15% punicalin, 5.08% punicalagin, 1.13% ellagic acid, and 0.27% gallic acid by weight (w/w).^13^ Several individual polyphenols have demonstrated TMA-reducing effects in other *in vitro* studies, including chlorogenic acid and gallic acid,^15^ caffeic acid, catechin, and epicatechin,^16^ and TMAO-reducing effects in animal models for resveratrol^17^ and chlorogenic acid.^18^ However, pomegranate polyphenols and their microbial metabolites, urolithins, had not been evaluated for their impact on TMA and TMAO formation. Furthermore, most of these existing studies focused on choline as the TMA precursor, which follows a different metabolic pathway than l-carnitine metabolism. This leaves the effects of polyphenols on l-carnitine-derived TMA production largely unexplored.

The production of the extract also included a spray drying step with gum Arabic as an excipient,^19,20^ which is an important methodological consideration for the identification of the main pomegranate extract constituent(s) responsible for the observed TMA-reducing effect. Gum Arabic is a soluble fibre which, like polyphenols, are poorly absorbed in the small intestine.^21^ Previous research has established that dietary fibre can inhibit TMA production,^22^ with diets high in soluble fibre reducing TMA and TMAO production in murine models by 40.6% and 62.6%, respectively.^23^ Therefore, gum Arabic must be considered as a potential bioactive contributor to the reduced l-carnitine metabolism previously observed when colon models were treated with pomegranate extract.

The microbial metabolism of pomegranate polyphenols, particularly ellagitannins and ellagic acid, results in the production of urolithins as primary metabolic end products (SI Fig. S1). These compounds, including urolithin A, urolithin B, and isourolithin A, represent the predominant pomegranate-derived metabolites detected in plasma, urine, and faecal samples.^24,25^ Current understanding suggests that urolithins are responsible for the health benefits attributed to pomegranates and other ellagitannin-rich foods.^26^ Consequently, determining whether urolithins contribute to the observed effects on TMA production becomes crucial for understanding the mechanism of action.

This research investigates how individual pomegranate polyphenols influence the microbial conversion of l-carnitine to γ-BB and TMA using a high-throughput *in vitro* model of the human colon.

## 2 Experimental

### 2.1 Materials

All solvents were at least high-performance liquid chromatography (HPLC) grade. Milli-Q ultrapure water was 18 MΩ cm resistivity. Trimethylamine hydrochloride (CAS 593-81-7), γ-BB (supplied under the commercial name 3-(carboxypropyl)trimethylammonium chloride, CAS 6249-56-5), urolithin A (CAS 1143-70-0), urolithin B (CAS 1139-83-9), trichloroacetic acid (TCA), glacial acetic acid, and heptafluorobutyric acid (HFBA) were procured from Merck. Fisher Scientific Limited supplied l-carnitine (CAS 541-15-1), ammonium acetate, piperazine-*N*,*N*′-bis(2-ethanesulfonic acid) (PIPES), and all other solvents used in this study. Isotopically labelled internal standards were sourced from multiple suppliers: l-carnitine-(trimethyl-d9) was acquired from Cambridge Isotope Laboratories (CAS 126827-79-0), trimethylamine-d9 hydrochloride from LGC Standards (CAS 18856-86-5), and γ-Butyrobetaine-d9 from Santa Cruz Biotechnology (CAS 479677-53-7). Individual pomegranate polyphenols were obtained from different suppliers: punicalagin was sourced from BOC Science (CAS 65995-63-3), punicalin from Apollo Scientific (CAS 65995-64-4), and Merck provided ellagic acid (CAS 476-66-4) and gallic acid (CAS 149-91-7). Gum Arabic (CAS 9000-01-5) was provided by Willy Benecke (Hamburg, Germany). The commercially available pomegranate extract (Dermogranate®) was purchased from Medinutrex (Catania, Italy).

### 2.2 Faecal donations

Faecal samples were obtained from participants enrolled in the QIB Colon Model Study (ClinicalTrials.gov ID NCT02653001). Ethical approval for this research was granted by the Human Research Governance Committee at Quadram Institute Bioscience (QIB) and the London-Westminster Research Ethics Committee (reference 15/LO/2169).

Participant recruitment focused on healthy adults of both sexes residing within ten miles of the Norwich Research Park who demonstrated regular bowel habits. Suitable donors exhibited average stool consistency between types 3 and 5 according to the Bristol Stool Chart.^27^ Exclusion criteria were: (i) being diagnosed with gastrointestinal disorders such as irritable bowel syndrome (IBS), inflammatory bowel disease (IBD), or coeliac disease; (ii) experiencing gastrointestinal symptoms including vomiting or diarrhoea within 72 hours preceding donation were excluded; (iii) recent medication use, specifically antibiotics or probiotics within four weeks of donation; (iv) being pregnant or lactating; (v) having undergone recent procedures requiring anaesthesia. In total, 8 donors made 22 donations.

Fresh faecal samples were collected on the same day as inoculation of the *in vitro* batch colon models to preserve microbial viability and metabolic activity. Sample processing was carried out within a class II microbiological safety cabinet (MSC). 10% w/v faecal slurries were prepared by combining fresh faecal sample with phosphate-buffered saline (PBS) in a 1 : 10 dilution ratio. Faecal slurries were homogenised using a Stomacher® 400 EVO at 230 revolutions per minute (rpm) for two 30-second intervals. No subject codes have been used in this manuscript.

### 2.3 Development of the high-throughput *in vitro* colon model

For the evaluation of individual polyphenols and other pomegranate extract constituents, the use of a small-scale, high-throughput colon model was necessary to measure TMA production efficiently and cost-effectively. The foundation for this approach came from previous work by Iglesias-Carres *et al.* (2021), who successfully screened various phytochemicals for their effects on TMA production from choline precursors.^15^ However, their original methodology lacked continuous pH monitoring, which represents a critical physiological factor in colonic fermentation models. Furthermore, the published method omitted glucose supplementation, whereas our previous studies investigating pomegranate extract utilised a medium with 1% glucose.^13^ Therefore, the high-throughput colon model was further optimised to be suitable for testing of pomegranate extract constituents.

The optimised colon model was carried out in 1.2 mL 96-well plates (LGC Genomics, Cat. No KBS-7001-130) within an anaerobic cabinet maintained at 37 °C to simulate human colonic conditions. Each well was inoculated with 2 mM l-carnitine alongside a final concentration of 1% faecal slurry derived from individual donors. The culture medium contained peptone water and yeast extract as nitrogen and carbon sources, supplemented with essential minerals including NaCl, K_2_HPO_4_, KH_2_PO_4_, MgSO_4_, NaHCO_3_, and CaCl_2_. l-Cysteine maintained reducing conditions necessary for obligate anaerobic bacteria. Additional components included bile salts to simulate colonic bile acid concentrations, hemin as a growth factor for certain bacterial species, Tween80 as a surfactant, and vitamin K_1_ to support bacterial metabolism. Buffering capacity was provided by the addition of PIPES buffer at 50 mM to enhance pH stability throughout the fermentation period. The pH of the medium was initially adjusted to 7.0 using drops of 10 M NaOH, bringing the PIPES buffer within its optimal buffering range where it maintains high solubility.^28^ After autoclaving, filter-sterilised d-glucose stock solution was added to achieve final concentrations of 0%, 0.1%, or 1.0%. The final pH was adjusted to 7.1 using drops of 0.5 M HCl or 0.5 M NaOH, after which the medium was placed in an anaerobic cabinet overnight to eliminate residual oxygen.

**Fig. 1:**
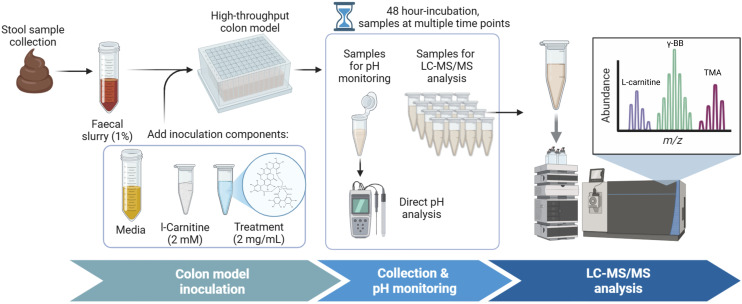
Flowchart of the experimental design. Created in BioRender.

For the inoculations, 792 µL sterile, pH-stable medium, 90 µL fresh faecal inoculum (1% final concentration), and 2 mM l-carnitine were added to each well (Fig. 1). Treatments were dissolved in PBS and added to the wells at 2 mg mL^−1^. Treatments included punicalagin, punicalin, ellagic acid, gallic acid, gum Arabic, urolithin A, and urolithin B. Additionally, initial optimisation experiments included the pomegranate extract at 22.8 mg mL^−1^ to test reproducibility from the batch colon model reported previously.^13^

For each experiment, an l-carnitine control was included, containing the faecal inoculum and l-carnitine substrate but excluding a treatment. A faecal control was also prepared containing only the faecal inoculum but excluding the l-carnitine substrate or treatments, which was used to account for background metabolite production from endogenous microbial processes. Samples designated for microbial metabolite quantification were collected over 48 hours and immediately stored at −80 °C until LC-MS/MS analysis was performed.

### 2.4 pH monitoring

Continuous pH monitoring throughout the fermentation period was achieved by measuring pH at 3–9 time points over 48 hours. At each time point, 75 µL was transferred from colon model wells into 0.6 mL microtubes. The pH was measured from the microtubes using an InLab Micro Pro-ISM electrode (Mettler Toledo, Leicester, UK) specifically designed for small-volume measurements. To prevent volume depletion from individual colon model wells, which could potentially alter fermentation conditions, pH measurements at each time point were obtained by alternating sampling between replicate wells for each donor.

### 2.5 Dosage information

The selection of 2 mg mL^−1^ concentration for individual pomegranate constituents was based on the manufacturer's Certificate of Analysis (CoA) (SI 2), which indicated that the punicalagin and ellagic acid (derivatives) in the extract are at least 7 and 10% (w/w), respectively. This corresponds to polyphenol quantities between 1.6–2.3 mg mL^−1^ when considering the high pomegranate dose (22.8 mg mL^−1^) reported previously.^13^ The polyphenol content of pomegranate juices has been shown to vary between 0.9 and 2.3 mg mL^−1^,^29,30^ making the doses used here physiologically relevant doses.

### 2.6 Sample preparation for LC-MS/MS

Quantification of l-carnitine, γ-BB, and TMA in collected samples was performed using liquid chromatography-tandem mass spectrometry (LC-MS/MS) following established protocols previously reported.^31^ Standards were prepared containing known concentrations of external standards (l-carnitine, γ-BB, and TMA) in a matrix containing 1% faecal inoculum and colon model media to match the matrix composition and account for potential matrix effects during analysis. The samples and standards were mixed with TCA to precipitate proteins and stop enzymatic activity. A mixture of deuterated (isotopically labelled) internal standards (l-carnitine-d9, γ-BB-d9, TMA-d9) prepared in 0.2 M aqueous acetic acid was added to each sample. Finally, samples and standards were diluted 1 : 20 with ultrapure water. All prepared samples and standards were transferred to capped analytical vials (Agilent Technologies) and analysed directly.

### 2.7 Metabolite quantification using LC-MS/MS

A description of the method used to quantify metabolites has been reported previously.^31^ The method includes targeted analysis using isotopically labelled internal standards (l-carnitine-d9, γ-BB-d9, TMA-d9). The LC-MS/MS analysis was carried out using a 1290 Infinity II LC System coupled to a 6490 Triple Quadrupole LC-MS system (Agilent Technologies) with an Acquity UPLC BEH C8 1.7 µm column (Waters, Massachusetts, United States). The mobile phase consisted of two solutions: 10 mM acetate and 0.05% HFBA dissolved in Milli-Q water (solution A), and 10 mM acetate and 0.05% HFBA (initially dissolved in 50 mL Milli-Q water) prepared in methanol (solution B).

### 2.8 Statistical analyses

l-Carnitine metabolism trajectories, and trajectories of metabolite formation, alongside pH measurements throughout the fermentation period, are presented as mean values ± SD across all donor samples. Statistical significance was established using an alpha level of *p* < 0.05 for all comparisons.

Linear mixed-effects models were employed to evaluate the effects of (pomegranate) polyphenols, urolithins, and gum Arabic on l-carnitine and metabolite concentrations. Random intercepts for individual donors were included to account for baseline differences between donors. Independent statistical models were fitted for each measurement at each time point. TMA concentrations measured in faecal controls (containing only microbial inoculum without l-carnitine substrate) were subtracted from all treatment conditions. This approach eliminates background TMA production from endogenous metabolite production. All metabolite concentrations are expressed as percentages of the initial l-carnitine concentration to facilitate comparison across different experimental conditions and donor samples.

Linear mixed-effects models were also employed for the pH data analysis, including fixed effects for treatment (*e.g.*, polyphenols), time point, and treatment × time interaction, and random intercepts for individual donors to account for repeated pH measurements over time. Time was treated as a categorical variable rather than continuous to enable precise estimation of treatment effects at each specific measurement time point.

*Post hoc* pairwise comparisons between each treatment condition and the controls were carried out at every time point using estimated marginal means (EMMs) such that time points with significant differences could be identified. For multiple comparison adjustments, a Dunnett's test was used to control for Type I errors (false positives) across all comparisons with the control condition.^32^

All graphs were generated using GraphPad Prism version 10.4.2, and R version 4.4.1 (R Core Team, 2024) in RStudio (Posit Software, PBC, Boston, MA). Statistical analyses were performed using RStudio with ‘lme4’,^33^ ‘lmerTest’,^34^ and ‘broom.mixed’^35^ packages for mixed modelling and ‘minpack.LM’^36^ for non-linear modelling.

## 3 Results

### 3.1 Development and testing of a high-throughput *in vitro* colon model

A high-throughput colon model was first optimised to ensure that it accurately reproduced physiologically relevant pH conditions and reflects the anaerobic metabolism of l-carnitine observed in the batch colon model^13^ (*i.e.*, to γ-BB and TMA), while maintaining pH within a physiologically relevant range: the desired pH range for the *in vitro* colon model ranges between 6.4–7.0, reflecting the physiological transition from transverse to distal colonic regions (transverse colon pH: 6.4–6.6; distal colon pH: 7.0).^37,38^ The development of the small-scale, high-throughput *in vitro* colon model used here was based on the methodology reported by Iglesias-Carres and colleagues (2021).^15^ The main adjustments that were tested were inclusion of glucose at 0.1 and 1.0% and the addition of a buffering agent (PIPES) at 50 mM.

**Fig. 2:**
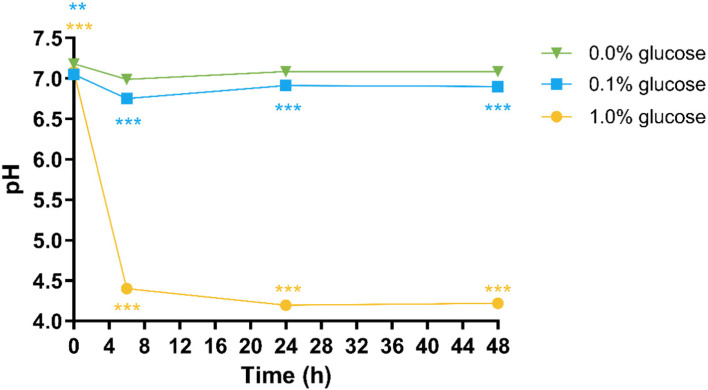
pH dynamics in the high-throughput colon model treated with varying glucose concentrations (0, 0.1, 1%) over a 48-hour fermentation period. Results are shown as mean values from triplicate measurements using a single donor sample (SDs were included in the graph but the variances are so low they are not visible). Statistical analysis employed linear mixed-effects models comparing glucose treatments against the control condition (0% glucose), including treatment as fixed effects with random intercepts for biological replicates (**p < 0.01, ***p < 0.001). *In vitro* colon models were inoculated with specified glucose concentrations (0%, 0.1%, or 1%) and 1% faecal inoculum from a healthy donor. Samples were collected throughout a 48-hour period with direct pH measurement using a InLab Micro Pro-ISM pH electrode.

Experiments done with 0.1% glucose added gave similar results in terms of bacterial cell viability (SI Fig. S2) and the pH remaining between 6.6–7.0 throughout the 48 hours of fermentation (Fig. 2) as were obtained when no glucose was added. The metabolism observed in the 0.1% glucose condition was similar to what was observed in the batch colon model.^13^

However, experimental conditions utilising 1% glucose resulted in pH values falling below physiologically relevant levels (Fig. 2). The pH reduction observed with 1% glucose coincided with substantial decreases in bacterial viability relative to glucose-free control conditions at both 24-hour (*p* = 0.031) and 48-hour (*p* = 0.004) time points, with viable cell populations falling below 1% of total bacterial counts at both time points (SI 2 and SI Fig. S2). In contrast, the moderate pH decline observed within the 0.1% glucose condition (Fig. 2) produced only slight reductions in microbial viability compared to the control (0% glucose) condition, maintaining 22% viable cells at 48 hours *versus* 28% in the control (*p* = 0.037) (SI 2 and SI Fig. S2). These findings indicate that increased glucose concentrations (∼1%) result in significant pH reductions accompanied by a substantial loss in bacterial viability.

Further testing was done using a colon model medium containing 0.1% w/v glucose and assessing whether we could reproduce the effects of the pomegranate extract observed in the batch colon model.^13^

**Fig. 3:**
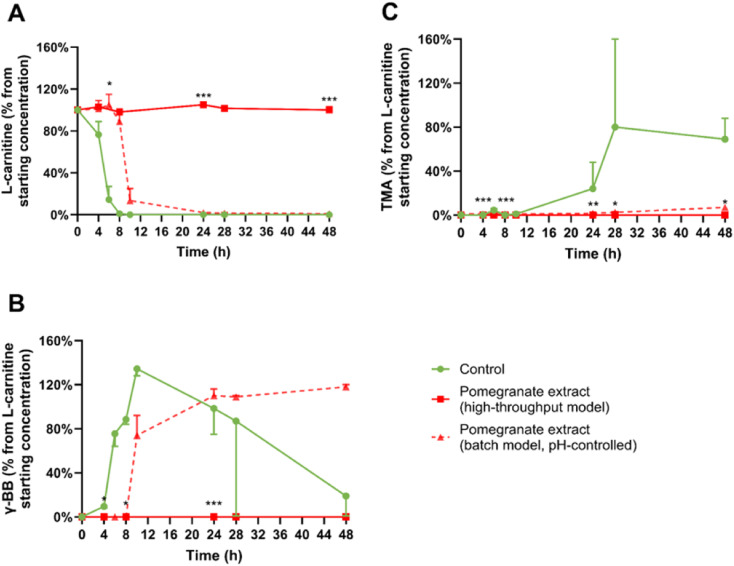
Effects of the pomegranate extract on l-carnitine metabolism *in vitro*. Average percentages of (A) l-carnitine, (B) γ-butyrobetaine (γ-BB), and (C) trimethylamine (TMA) relative to initial l-carnitine concentration are displayed over 48 hours. Results are shown as mean ± SD from 2 donors with 1–2 biological replicates per donor. Statistical analysis employed linear mixed models, including treatment as fixed effects with random intercepts for donors (*p < 0.05, **p < 0.01, ***p < 0.001), to measure significant differences between the pomegranate extract (high-throughput model) and the control. High-throughput and batch colon models were inoculated with 1% faecal inoculum from a healthy donor, 2 mM l-carnitine, and the pomegranate extract. After collection, samples were directly stored at −80 °C until LC-MS/MS quantification using isotope-labelled internal standards. The dashed line indicates the results obtained for treatments at a comparable dose of pomegranate extract (22.8 mg mL^−1^) and l-carnitine but in a pH-controlled *in vitro* batch colon model, as described in a previously published report.^13^

Inclusion of the pomegranate extract (22.8 mg mL^−1^) caused a complete inhibition of l-carnitine conversion to γ-BB (Fig. 3A and B), and, as expected, no accumulation of TMA (Fig. 3C). This effect likely resulted from severe pH reduction to suboptimal levels of 4.9 by 48 hours, substantially below the target range of 6.4–7.0, in pomegranate extract-treated experimental conditions (Fig. 6; although Fig. 6 appears later in the manuscript, we refer to it here because it summarises all pH measurements across experiments in one place). However, treatment with several isolated polyphenols did not cause the pH to be reduced below the target range, which means the high-throughput model is suitable for testing the effects of isolated polyphenols (the results are presented in the next section).

### 3.2 The effects of individual constituents of the pomegranate extract on l-carnitine metabolism

Experiments were performed to investigate the effects of the known components of the pomegranate extract on anaerobic l-carnitine metabolism. These were the pomegranate polyphenols punicalagin, punicalin, ellagic acid, and gallic acid, the ellagitannin/ellagic acid microbial metabolites urolithin A and urolithin B, and the spray-drying excipient gum Arabic (SI 2).

**Fig. 4:**
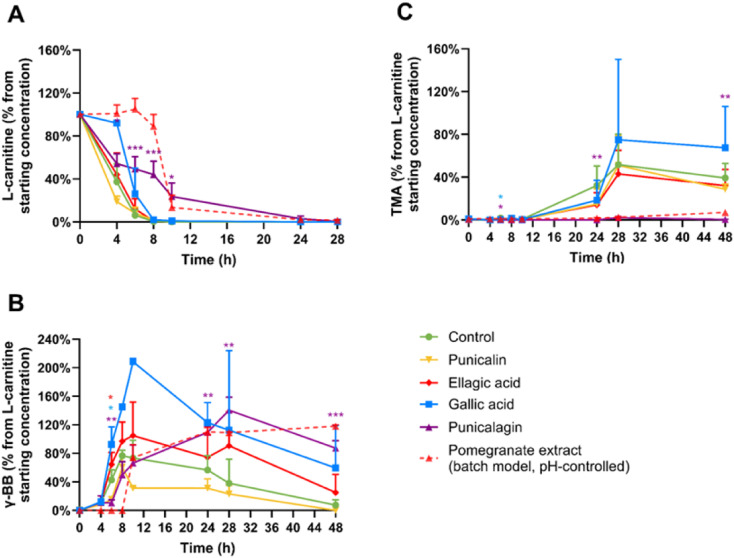
Effects of pomegranate polyphenols and gum Arabic at 2 mg mL^−1^ on *in vitro*l-carnitine metabolism. Average percentages of (A) l-carnitine, (B) γ-butyrobetaine (γ-BB), and (C) trimethylamine (TMA) relative to initial l-carnitine concentration are displayed over 48 hours. Results are shown as mean ± SD from 2–5 donors with 1–4 biological replicates each. Statistical analysis employed linear mixed models, including treatment as fixed effects with random intercepts for donors (*p < 0.05, **p < 0.01, ***p < 0.001), to measure significant differences between the polyphenol treatments (high-throughput model) and the control. High-throughput and batch colon models were inoculated with 1% faecal inoculum from a healthy donor, 2 mM l-carnitine, and the treatment. After collection, samples were directly stored at −80 °C until LC-MS/MS quantification using isotope-labelled internal standards. The dashed line indicates the results obtained for treatments at a comparable dose of pomegranate extract (22.8 mg mL^−1^) and l-carnitine but in a pH-controlled *in vitro* batch colon model, as described in a previously published report.^13^

No substantial or statistically significant effects were observed for punicalin, ellagic acid, gallic acid, urolithins, or gum Arabic. But, punicalagin significantly suppressed the conversion of l-carnitine to γ-BB (Fig. 4). At 8 and 10 hours of fermentation, respectively 43.6 and 23.6% of the l-carnitine was still remaining within the punicalagin-treated condition, compared to 1 and 0% of the l-carnitine remaining in the control condition (*p* < 0.001). Furthermore, punicalagin almost entirely blocked TMA production, yielding only 0.4% TMA from the initial l-carnitine at 48 hours *versus* 39.2% TMA production in the control condition (*p* = 0.003). These results demonstrate that punicalagin is particularly effective against the secondary conversion step in the l-carnitine → γ-BB → TMA metabolic pathway. Punicalagin's inhibitory effects remained consistent across all individual donors (SI Fig. S3).

**Fig. 5:**
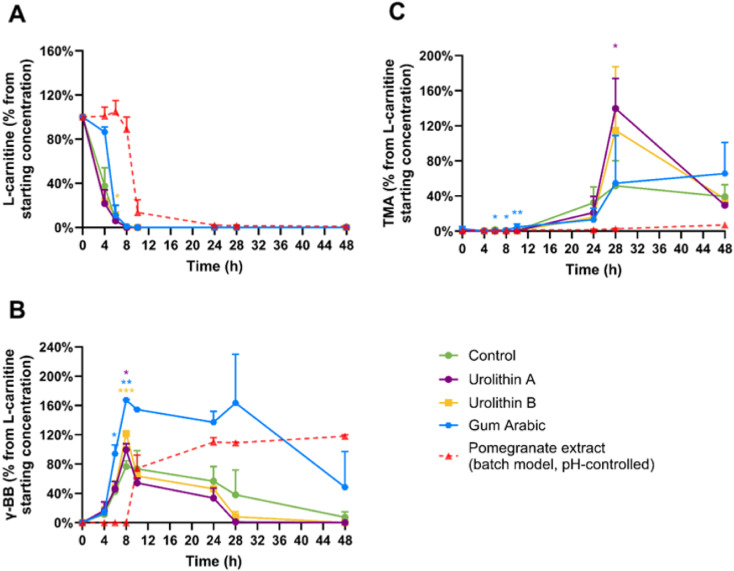
Effects of urolithins A and B at 2 mg mL^−1^ on *in vitro*l-carnitine metabolism. Average percentages of (A) l-carnitine, (B) γ-butyrobetaine (γ-BB), and (C) trimethylamine (TMA) relative to initial l-carnitine concentration are displayed over 48 hours. Results are shown as mean ± SD from 3–5 donors with 1–4 biological replicates each. Statistical analysis employed linear mixed models, including treatment as fixed effects with random intercepts for donors (*p < 0.05, **p < 0.01, ***p < 0.001), to measure significant differences between the urolithins and gum Arabic (high-throughput model) *versus* the control. High-throughput and batch colon models were inoculated with 1% faecal inoculum from a healthy donor, 2 mM l-carnitine, and the treatment. After collection, samples were directly stored at −80 °C until LC-MS/MS quantification using isotope-labelled internal standards.

Neither urolithin A or urolithin B significantly inhibited TMA formation from l-carnitine (Fig. 5). In fact, there is evidence that urolithin B, and particularly urolithin A, increased the rate of conversion of γ-BB to TMA, with urolithin A significantly increasing TMA concentrations at 28 hours compared with the control (*p* = 0.021) and urolithin B showing a non-significant trend (*p* = 0.088). These findings suggest that urolithins did not play a role in the inhibitory effect of the pomegranate extract on the conversion of l-carnitine to γ-BB and TMA.

### 3.3 Changes in pH during high-throughput colon model fermentations

**Fig. 6:**
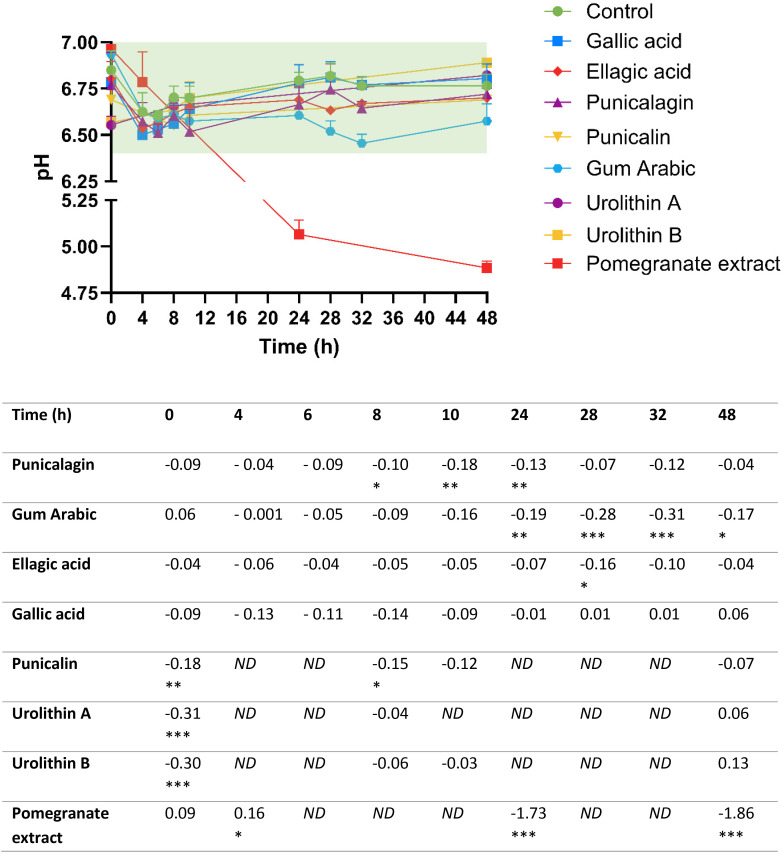
pH trajectories in high-throughput *in vitro* colon models treated with the pomegranate extract, individual extract constituents and metabolites, over 48 hours. The target pH range of 6.4–7.0 is highlighted in green. Results are shown as mean ± SD of 2–7 donors. Results in the table are shown as mean pH decrease relative to control. Statistical analysis employed linear mixed-effects models comparing treatments to control at individual time points, with treatment and time as fixed effects and donors as random intercepts (*p < 0.05, **p < 0.01, ***p < 0.001). High-throughput *in vitro* colon models were inoculated with 1% faecal inoculum from a healthy donor, 2 mM l-carnitine, and the treatment (22.8 mg mL^−1^ pomegranate extract and 2 mg mL^−1^ for all other treatments). Samples were collected throughout a 48-hour period with direct pH measurement using a InLab Micro Pro-ISM pH electrode. ND, not determined.

Most treatment conditions resulted in initial pH decreases of 0.2–0.3 units between baseline and 4 hours of fermentation, with the exception of urolithin treatments, which commenced at reduced baseline pH values (Fig. 6). Following this initial decline, pH values for all treatments slightly increased and were maintained between pH 6.7–6.8, except for the gum Arabic treatment, which continued declining but remained above the minimum threshold of pH 6.4. Overall, these data show that when using the high-throughput colon model with added buffer salts (50 mM PIPES), the pH remains in the target range (6.4–7.0) in all fermentations treated with isolated constituents, but not in fermentations with the pomegranate extract.

## 4 Discussion

We recently reported that a pomegranate extract could dose-dependently inhibit the production of γ-BB from l-carnitine and inhibit the production of TMA.^13^ Therefore, the main objective of this study was to identify the specific component(s) within the pomegranate extract that are responsible for the reported effects of the pomegranate extract on l-carnitine metabolism. Since human faecal bacteria can convert ellagitannins/ellagic acid to urolithins, the main urolithins observed in humans were also tested (urolithins A and B). The key finding from the current study was that amongst all phenolic and non-phenolic pomegranate extract constituents that were tested, punicalagin alone could significantly reduce TMA production from l-carnitine. In fact, punicalagin almost completely blocked TMA production, thereby establishing this compound as the only bioactive component within the pomegranate extract that is responsible for the inhibitory effects on l-carnitine metabolism that were previously documented by the authors.^13^

Punicalagin has gained substantial scientific attention due to its beneficial properties, particularly its favourable impacts on the gut microbiome composition, including the promotion of beneficial host-microbiota interactions and restoration of microbial balance following dysbiosis.^39^ The beneficial effects of punicalagin have been partially credited to its microbial metabolites, ellagic acid and urolithins.^25,40^ Moreover, there are numerous reports showing that the health benefits of pomegranate depend on the urolithin metabotype.^41–43^ Meanwhile, the current study demonstrated that neither ellagic acid nor urolithins could significantly suppress l-carnitine metabolism and TMA formation, indicating that the observed effects are exclusively attributable to punicalagin itself rather than its metabolic derivatives. These results suggest that certain health benefits to pomegranate consumption may be solely dependent on punicalagin and not dependent on urolithins or individual urolithin metabotype. Hence, the extent of punicalagin's bioactivity may depend on the proportion that reaches the colon, which in turn is influenced by its rate of hydrolysis to ellagic acid in the stomach and small intestine.^44,45^

Punicalagin exhibits limited stability in gastrointestinal conditions; studies in animal models indicate that only a small fraction remains intact, with most undergoing hydrolysis and microbial metabolism before reaching the colon.^46,47^ Given that the observed effects are attributable to punicalagin itself rather than its metabolites, it may be useful to develop strategies that can enable more of the intact compound to reach the colon. Approaches such as encapsulation or formulation with protective carriers can enhance stability and limit hydrolysis in the upper gastrointestinal tract.^48,49^ Besides, targeted delivery approaches including pH-sensitive and bacteria-degradable polymer coatings, bacteria-degradable prodrugs, and bacteria-degradable hydrogels or matrix systems, can offer promising strategies towards site-specific release within the colon.^50^ Materials such as chitosan, sodium alginate, pectin, and guar gum have shown promise for protecting and targeting phenolic compounds to the colon, owing to their pH-responsive and enzyme-degradable properties.^51^

Although it was not the purpose of this research to establish the mechanisms by which the pomegranate polyphenol punicalagin inhibits the conversion of l-carnitine to TMA, a number of plausible mechanisms can be proposed. These include: (i) decreased abundance of microorganisms harbouring the genes necessary for l-carnitine metabolism, (ii) suppression of transcriptional activity of these genes, (iii) inhibition of proteins (including enzymes and transporter proteins) that facilitate the conversion of l-carnitine to γ-BB and γ-BB to TMA, and (iv) binding of the substrates (l-carnitine, γ-BB) such that the ability of microbes to metabolise them is slowed down or completely inhibited.

Firstly, polyphenols may alter gut microbial composition or activity through their ‘duplibiotic’ properties,^52,53^*i.e.*, simultaneously antimicrobial and growth-stimulating effects that depend on the specific compound and microorganism. Previously, we have shown that the pomegranate extract did not reduce the overall microbial viability.^13^ However, punicalagin may selectively suppress the bacteria responsible for the conversion of l-carnitine to γ-BB and TMA, such as *Escherichia coli*,^54,55^ whilst promoting beneficial species like *Bifidobacterium*, *Lactobacillus*, and *Akkermansia muciniphila*.^56–58^

Secondly, punicalagin might affect the expression of the genes involved in microbial l-carnitine and γ-BB metabolism pathways. Polyphenols can interact with transcription factors or regulatory enzymes^59,60^ and alter cellular redox status, which then regulates microbial metabolism through redox-sensor transcription factors.^61,62^ These redox changes could shift expression or functionality of enzymes in the l-carnitine metabolic pathway.

Thirdly, pomegranate polyphenols may directly disrupt the microbial proteins involved in l-carnitine and γ-BB metabolism, including membrane transporters or intracellular enzymes. Some polyphenols bind to membrane proteins like porins, altering permeability and restricting substrate access to enzymes.^63,64^ By limiting l-carnitine or γ-BB entry into cells or interaction with intracellular enzymes, polyphenols may reduce TMA production efficiency.

Finally, a possibility specific to ellagitannins (such as punicalagin and punicalin) involves their capacity to bind nitrogenous compounds, including choline, l-carnitine, γ-BB, and TMA.^65^ For instance, tannic acid has been reported to form non-covalent complexes with choline.^66^ Punicalagin possesses numerous hydroxyl groups and aromatic ring structures (SI Fig. S1), enabling hydrogen bond donation to polar sites and formation of cation–π interactions.^65,67^l-Carnitine is a zwitterion, containing both positive and negative charges, and exhibits high polarity. The multiple polar sites within l-carnitine may be able to establish hydrogen bonds with punicalagin hydroxyl groups. Additionally, the positive charge on the trimethylammonium group of l-carnitine can conceivably form cation–π interactions with punicalagin's aromatic rings. Consequently, punicalagin may have inhibited l-carnitine metabolism through non-covalent binding interactions with l-carnitine and γ-BB, making these substrates less available to microbial enzymes and preventing TMA formation. In contrast to punicalagin, punicalin is a smaller molecule (782 Da *versus* punicalagin's 1084 Da) and has reduced hydrogen bonding potential, with punicalin possessing approximately 12 hydrogen donors and 21 acceptors compared to punicalagin's 17 hydrogen donors and 30 acceptors (considering all hydroxyl and carbonyl groups). Furthermore, punicalagin contains a greater number of aromatic rings than punicalin, increasing its propensity for cation–π interaction formation. Therefore, larger ellagitannins with enhanced hydroxyl group density and aromatic ring content (punicalagin > punicalin) may demonstrate greater potential for l-carnitine binding, potentially explaining why inhibitory effects on l-carnitine metabolism were observed for punicalagin but not punicalin. The data presented here suggest that a reduced pH can influence l-carnitine metabolism. These observations are consistent with those in a previously published report concerned with use of an *in vitro* batch colon model to study methylamine metabolism by human faecal microbiota.^31^ It was shown that, in pH-controlled batch fermentations of human colon models, TMA was produced from all three dietary precursors, l-carnitine, choline, and betaine, in addition to the metabolic intermediate γ-BB, replicating the metabolic pathways observed in humans. In contrast, no TMA formation occurred when fermentations were performed without pH control, highlighting the critical role of pH in regulating TMA production.^31^ Here, we report that the pomegranate extract produced the most substantial pH decrease, despite the inclusion of 50 mM PIPES buffer to enhance buffering capacity. Concomitantly, the conversion of l-carnitine to γ-BB and TMA was completely blocked by the pomegranate extract in the high-throughput colon model, suggesting that the pH decline was most probably responsible for the inhibited l-carnitine metabolism. This corresponds to the finding that a glucose concentration of 1% substantially reduced the pH, well below the target pH of 6.4–7.0, which resulted in a reduced microbial viability of viable cell populations falling below 1% of total bacterial counts at 48 hours of fermentation. Changes in pH can substantially modify gut microbiome composition, with a reduced pH resulting in a decreased microbial community structure and abundance of specific bacterial taxa.^68^ Microbial metabolism, growth patterns, and enzymatic activity are influenced by multiple factors including environmental pH.^69^ Microbial growth becomes compromised when environmental pH deviates from the pH for optimal growth. For example, research has demonstrated that a single unit pH decrease can reduce microbial metabolic activity by up to 50%.^69^ Hence, a pH reduction in the high-throughput colon model below the target range of 6.4–7.0 may result in a reduced microbial viability, and therefore the inhibition of l-carnitine metabolism.

A limitation of this work involves the incompatibility of the pomegranate extract with the high-throughput colon model due to the substantial pH decline. Therefore, the effects of individual pomegranate constituent effects on microbial l-carnitine metabolism could not be directly compared with the pomegranate extract as a positive control. Nevertheless, punicalagin achieved complete TMA inhibition, suggesting that punicalagin's effect exceeds that observed for the complete pomegranate extract. The pH decline within the high-throughput colon model likely results from constituents other than the polyphenols present in the extract. Gum Arabic, which was utilised in the spray-drying process of the extract, represents a soluble fibre metabolised by gut microbiota into organic acids, including short-chain fatty acids (SCFAs) that correlate with reduced colonic pH.^70^ However, although gum Arabic alone led to a reduced pH, it did not fully account for the pH reduction observed following pomegranate extract treatment. The substantial pH decline might be better explained by the carbohydrates in the extract, which comprises a significant proportion (90–95%) of the extract's macronutrient composition (SI 2). Studies employing *in vitro* colon models demonstrated that carbohydrate utilisation leads to SCFA production and pH reduction.^71^ As such, carbohydrate-rich products may not be compatible with the high-throughput colon model for the evaluation of their effects on l-carnitine metabolism. This work also emphasises the importance of continuous pH monitoring throughout experiments when utilising colon models lacking pH control systems.

When estimating γ-BB and TMA concentrations, some of the metabolites exceeded 100% of the initial l-carnitine concentration. These discrepancies may have resulted from matrix effects. Matrix effects are common phenomena referring to the effects of components in the sample matrix on analyte ionisation (*e.g.*, γ-BB, TMA), subsequently affecting MS-based quantity estimations.^72^ Here, the standard curve was prepared in a matrix consisting of colon model medium and 1% faecal slurry, reflecting the baseline experimental matrix without any treatments. Hence, the standard curve matrix cannot accurately represent sample matrices at later experimental time points or following treatment addition. This can result in matrices containing microbial metabolites including SCFAs and phenolic catabolites. These metabolites can affect the LC-MS/MS quantification through ion suppression or enhancement. Essentially, the matrix undergoes constant temporal changes and differs in each colon model vessel. Therefore, to achieve matrix-matched standards, separate standard curves would need to be prepared for each time point and treatment, which is impractical.

The present study did not assess microbial community composition. Although our previous work demonstrated that a punicalagin-rich pomegranate extract did not reduce overall microbial viability,^13^ we cannot determine from the current dataset whether punicalagin alters the abundance of taxa involved in l-carnitine or γ-BB metabolism. Ongoing metagenomic and functional analyses will address these questions; however, such investigations were beyond the scope of the current study.

In our previous work using a punicalagin-rich pomegranate extract, we observed inhibition of TMA formation from both l-carnitine and, to a lesser extent, choline.^13^ Because the metabolic pathways and microbial genes involved in l-carnitine metabolism differ from those used for choline and betaine, the current study focused specifically on identifying the extract constituent responsible for inhibiting the l-carnitine → γ-BB → TMA pathway. The present findings indicate that punicalagin is the primary inhibitor of l-carnitine metabolism from the pomegranate extract. Consequently, it is likely that punicalagin will be the compound in the pomegranate extract responsible for inhibiting the conversion of choline to TMA, although this will need to be tested experimentally in future studies. Having demonstrated that punicalagin alone can completely inhibit the TMA production from l-carnitine, future research should furthermore focus on through which mechanisms punicalagin does this (*e.g.*, reduced *cai* abundance and expression, reduced microbial protein activity, binding of substrates) and to test if a pomegranate extract and/or punicalagin can also inhibit circulating TMAO levels in a human intervention study.

## 5 Conclusion

Here it was shown that, of all the phenolic and non-phenolic components of the pomegranate extract, punicalagin is the principal inhibitor of microbial l-carnitine metabolism, markedly suppressing γ-BB formation and almost completely abolishing TMA production in high-throughput *in vitro* colon models. Hence, punicalagin can be identified as a promising candidate for dietary modulation of the TMA/TMAO pathway, with potential implications for reducing cardiovascular diseases risk. Further studies are warranted to explore the underlying mechanisms by which punicalagin inhibits the microbial l-carnitine metabolism to TMA and human intervention studies will be essential to validate these findings.

## Author contributions

Conceptualisation, J. E. H. and P. A. K.; methodology, J. E. H., M. I. G. E., and D. L.; formal analysis, visualisation, and writing – original draft, J. E. H.; writing – review and editing, M. I. G. E. and P. A. K.; supervision, P. A. K.; funding acquisition, P. A. K. All authors have read and agreed to the published version of the manuscript.

## Conflicts of interest

There are no conflicts to declare.

## Abbreviations

CVD: Cardiovascular disease
EMM: Estimated marginal means
FMO: Flavin-dependent monooxygenase
γ-BB: γ-Butyrobetaine
HFBA: Heptafluorobutyric acid
IBD: Inflammatory bowel disease
IBS: Irritable bowel syndrome
LC-MS/MS: Liquid chromatography-tandem mass spectrometry
MSC: Microbiological safety cabinet
PBS: Phosphate-buffered saline
PIPES: Piperazine-*N*,*N*′-bis(2-ethanesulfonic acid)
rpm: Revolutions per minute
SCFA: Short-chain fatty acid
TCA: Trichloroacetic acid
TMA: Trimethylamine
TMAO: Trimethylamine *N*-oxide

## Supplementary Material

FO-017-D5FO04781A-s001

## Data Availability

The data that support the findings of this study are openly available in Zenodo at https://doi.org/10.5281/zenodo.17296136. Supplementary information (SI) is available. The SI includes a schematic of punicalagin metabolism, technical certificates for the pomegranate extract used, flow cytometry methodology and aviability data from the high-throughput colon models, and the individual *in vitro*l-carnitine metabolism trajectories per donor. See DOI: https://doi.org/10.1039/d5fo04781a.
