# Supplementary material for: Punicalagin is the key pomegranate polyphenol inhibiting gut microbial trimethylamine (TMA) production from l-carnitine in an in vitro human colon model
Source: Food Funct. 2026 Feb 2;17(4):1827–40. doi: 10.1039/d5fo04781a (PMC12863279; doi:10.1039/d5fo04781a)
Supplement: FO-017-D5FO04781A-s001 [file FO-017-D5FO04781A-s001.pdf]

Supplementary Information (SI) for Food & Function.
This journal is © The Royal Society of Chemistry 2026

Supplementary Information

Punicalagin is the Key Pomegranate Polyphenol Inhibiting Gut Microbial Trimethylamine (TMA) Production from L-Carnitine in an In Vitro Human Colon Model

Authors: Haarhuis, J.E. [a], Gamal El-Din, M.I. [a, b], Lamprinaki, D. [a], Kroon, P.A. [a] *

[a] Quadram Institute Bioscience, Research Park, Rosalind Franklin Rd, Norwich NR4 7UQ, UK

[b] Department of Pharmacognosy, Faculty of Pharmacy, Ain-Shams University, Abbasia, Cairo, 11566, Egypt

* Corresponding author.

Supplement 1

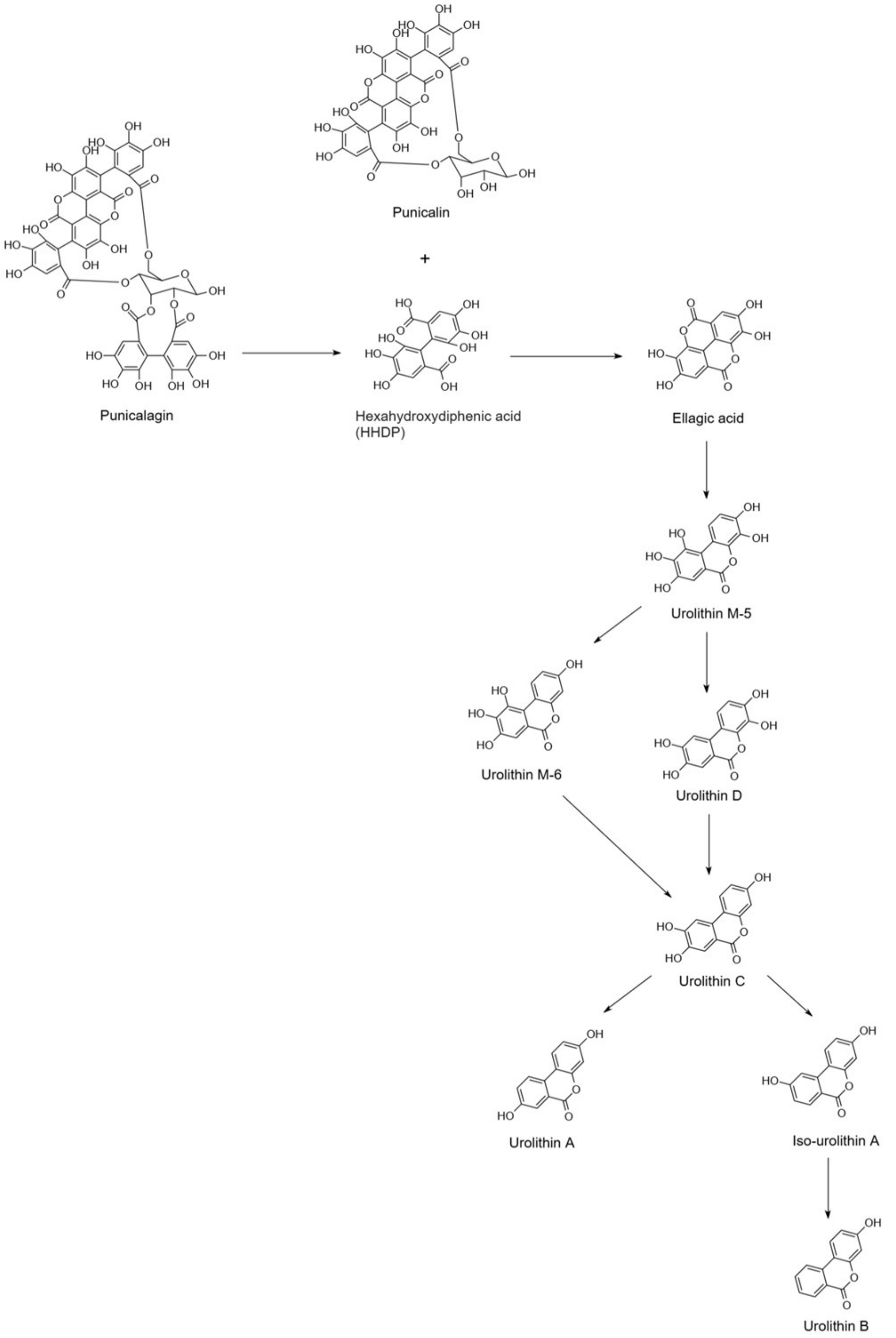

Supplementary Figure S1. Schematic overview of the breakdown of punicalagin to urolithins. *In the human gut, punicalagin is first converted to punicalin and subsequently to ellagic acid. Within the colon, ellagic acid is converted to various urolithins by the gut microbiota.*

Supplement 2

Pomegranate (Dermogranate®) extract Certificate of Analysis (CoA) and Technical Data Sheet (TDS) obtained from Medinutrex (Sicily, Italy).

CERTIFICATE OF ANALYSIS	
medinutrex mediterranean nutraceutical extracts	Product: DERMOGRANATE™
E-mail: info@medinutrex.com	Compilation date: 05.06.2023<br>Last review: 11.09.2023

Batch n°	
Manufacture date	05.06.2023
Expiry date	05.06.2026
Storage	Keep container unopened in cool, dry place
Country of origin	Italy
Botanical name	Punica granatum L.
Plant part used	fruit

PHYSICAL/CHEMICAL TEST

Analysis	Specification	Results
Total polyphenols (w/w)	≥20.0	Conform
Ellagic acid and derivatives (w/w)	≥10.0	Conform
Punicalagins (w/w)	≥7.0	Conform
Form	Powder	Conform
Color	Brown	Conform
Taste	Typical of pomegranate	Conform
Smell	Typical of pomegranate	Conform
pH	> 3.0	Conform
Solubility	Moderately soluble in water	Conform

Lead (Pb)	< 3ppm	Conform
Arsenic (As)	< 3ppm	Conform
Cadmium (Cd)	< 1ppm	Conform
Mercury (Hg)	< 0.1ppm	Conform
Pesticides	Conform to Reg CE 396/2005	Conform
PAH	Conform to Reg CE 1881/2006	Conform
Benzo(a)pyrene	< 10 µg/kg	Conform
Sum of Benzo(a)pyrene, Benz(a)anthracene, Benzo(b)fluoranthene and Chrysene	< 50 µg/kg	Conform
Residual solvent (ethanol)	< 0.2%	Conform
Total plate count (CFU/g)	< 10000	Conform
Yeast and Mould (CFU/g)	< 100	Conform
Enterobacteriaceae (CFU/g)	< 100	Conform
Escherichia coli	Absent	Conform
Pseudomonas aeruginosa	Absent	Conform
Staphylococcus aureus	Absent	Conform
Salmonella specie	Absent	Conform
Particle size	More than 90% pass 300 µm	Conform

GMO: product no- GMO (Reg. 1829/2003-1830/2003 EC).

Irradiations: this product has not been irradiated.

TECHNICAL DATASHEET	
medinutrex<br>mediterranean nutraceutical extracts<br>www.medinutrex.com	Product:<br>DERMOGRANATE™
E-mail: info@medinutrex.com	Compilation date: 05.06.2023<br>Last review: 09.06.2023

PART 1: GENERAL INFORMATION	
Product name	**Dermogranate**
Botanical name	*Punica granatum* L.
INCI name	Punica Granatum Fruit Extract
CAS number	84961-57-9
Extraction solvent	ethanol / water
DER	up to stated assay
Excipients quantity	arabic gum max 20.0%
Botanical family	Punicaceae
Origin of the raw material used for this product	Italy, Sicily
Growing condition	cultivated
Vegetative period	at maturity
Collection period	September - November
Part of plant used	fruit
Preparation type	dry extract
Particle size	not less than 90% through 300 microns
Intended Use	Raw material for food and cosmetics.
Active substances of the plant	polyphenols, ellagic acid and derivatives, punicalagins
Biological marker	ellagic acid

Radioactivity	< 600 Bq/kg
Contra-indications, warnings	ellagic acid (as a tannin) could cause precipitation of proteins
Eventual particular notes	none
Nutritional values	Carbohydrates: 90-95% - Fat: 0-1% - Protein: 0-1% - Minerals: 3-5%<br>Energy value (Kcal/100 gr): 409<br>Energy value (KJ/100 gr): 1711
Preservatives	absent
Antioxidants	absent
Storage conditions	store in a well closed container away from moisture and direct sun light
Retest date	three years

PART 2: TECHNICAL SPECIFICATIONS	
Product	Dermogranate
Code	
Description	hygroscopic powder
Colour	Brown
Odor	characteristic
Taste	characteristic
Assay: Total Polyphenols (met. Spectrophotometric)	≥ 20.00 % w/w
Assay: Ellagic acid and derivatives (met. HPLC)	≥ 10.00 % w/w
Assay: Punicalagins (met. HPLC)	≥ 7.00 % w/w
Bulk density	450 - 650 g/l
Loss on drying	≤ 5.0 % w/w
pH	3.0 – 5.0
Hydrosolubility	partially watersoluble
Heavy metals	< 20 ppm (method C Ph. Eur. current edition)
Lead (ref. Reg. (EC) 1881/2006)	≤ 3.0 ppm*
Cadmium (ref. Reg. (EC) 1881/2006)	≤ 1.0 ppm*

Mercury (rif. Reg. (EC) 1881/2006)	≤ 0.1 ppm*
Residual solvents	complies to Ph. Eur. current edition and Directive 2009/32/EC*
Pesticides	complies to Ph. Eur. current edition and Reg. 2005/396/EC and amendments concerning pesticides residues searched (with reference to E/D ratio)* (conformity reported in the certificate of analysis if available and/or upon request)
Aflatoxins	Aflatoxin B1: < 2 ppb*<br>Aflatoxin B1,B2,G1,G2: < 4 ppb*
Benzo(a)pyrene (Reg. (EC) 1881/2006)	≤ 10 ppb*
Sum of Benzo(a)pyrene, benzo(a)anthracene, benzo(b)fluoranthene and chrysene (Reg. (EC) 1881/2006)	≤ 50 ppb*
Microbiological quality (Ref. Ph. Eur. current edition depending on the intended use)	
- Bacterial count (TAMC: ref. 5.1.8, cat. B oral use)	≤ 10 000 ufc/g
- Yeasts and Moulds (TYMC: ref. 5.1.8, cat. B oral use)	≤ 100 ufc/g
- Pathogens (ref. 5.1.8, cat. B oral use)	Salmonella: absent in 25 g*<br>Escherichia coli: absent in 1 g*
Bile-tolerant gram-negative bacteria (ref. 5.1.8, cat. B oral use)	≤ 100 ufc/g

NOTE
GMO: Free from GMO (Reg. (EC) 1829/2003 and 1830/2003)<br><br>BSE/TSE FREE - GLUTEN FREE<br><br>ALLERGENS: Free from substances or products causing allergies or intolerances<br><br>NANOMATERIALS: Free from engineered nanomaterials<br><br>(Reg. (EU) 1169/2011 Annex II)<br><br>Melamine Free (Reg. (EU) 594/2012)
THIS PRODUCT AND RAW MATERIAL, FROM WHICH IT IS OBTAINED, SHOULD NOT BE IRRADIATED
THIS PRODUCT IS SUITABLE FOR OVO-LACTO-VEGETARIANS AND VEGANS
The data reported in this Technical Data Sheet (excluding analytical ones) are taken from literature, among which (if applicable) Italian ministerial guidelines for physiological effects and CosIng (European Commission database for information on cosmetic substances).<br><br>In any case this information will not discharge you from duty to identify and to monitor the product according to the use to which it is intended and the current legislation in the Country of use.<br><br>(*) analysis performed on basis of specific self-control plan<br><br>- The drug extract ratio (DER) is intended as drug (final) extract ratio, included any excipient, as per Ph. Eur. current edition, monograph n. 52300 "Monographs on herbal drug extracts"<br><br>- Herbal extracts with assay are intended as standardised extract, unless otherwise specified<br><br>- In standardised extract the excipient is added for adjust the content of constituent(s) (assay), in extracts based on DER to guarantee the final DER

Supplement 2: Use of flow cytometry to estimate bacterial cell viability

Methods

High-throughput colon models were carried out in 15 mL falcon tubes (Starlab Ltd, Milton Keynes, UK) in an anaerobic cabinet maintained at 37°C to simulate human colonic conditions. The culture medium contained peptone water, yeast extract, NaCl, K_2HPO_4, KH_2PO_4, $MgSO_4$, $NaHCO_3$, $CaCl_2$, L-Cysteine, bile salts, hemin, Tween80, and vitamin K_1. Buffering capacity was provided by the addition of 50 mM PIPES buffer. Filter-sterilised D-glucose stock solution was added after autoclaving to final concentrations of 0%, 0.1%, and 1.0%. The final pH was adjusted to 7.1 using drops of 0.5 M HCl or 0.5 M NaOH, after which the medium was placed in an anaerobic cabinet overnight to eliminate residual oxygen. Each tube was inoculated with a final concentration of 1% faecal slurry derived from one individual donor. Samples were collected over 48 hours and immediately prepared for flow cytometry analyses.

After collection, samples were filtered through a pluriStrainer Mini 40 µm (pluriSelect Life Science, Leipzig, Germany). A dye was prepared using Guava® Bacterial Count & Viability Kit (Luminex, Austin, TX, USA) and samples were diluted 1:10. Prior to analysis, samples were vortexed and incubated at 37 °C for 30 minutes. Samples were loaded on an ImageStream^X Mk II (Cytek Biosciences, Fremont, CA, USA) imaging flow cytometer and analysed using IDEAS software version 6.2 (Cytek Biosciences) to determine bacterial cell counts and viability.

Results

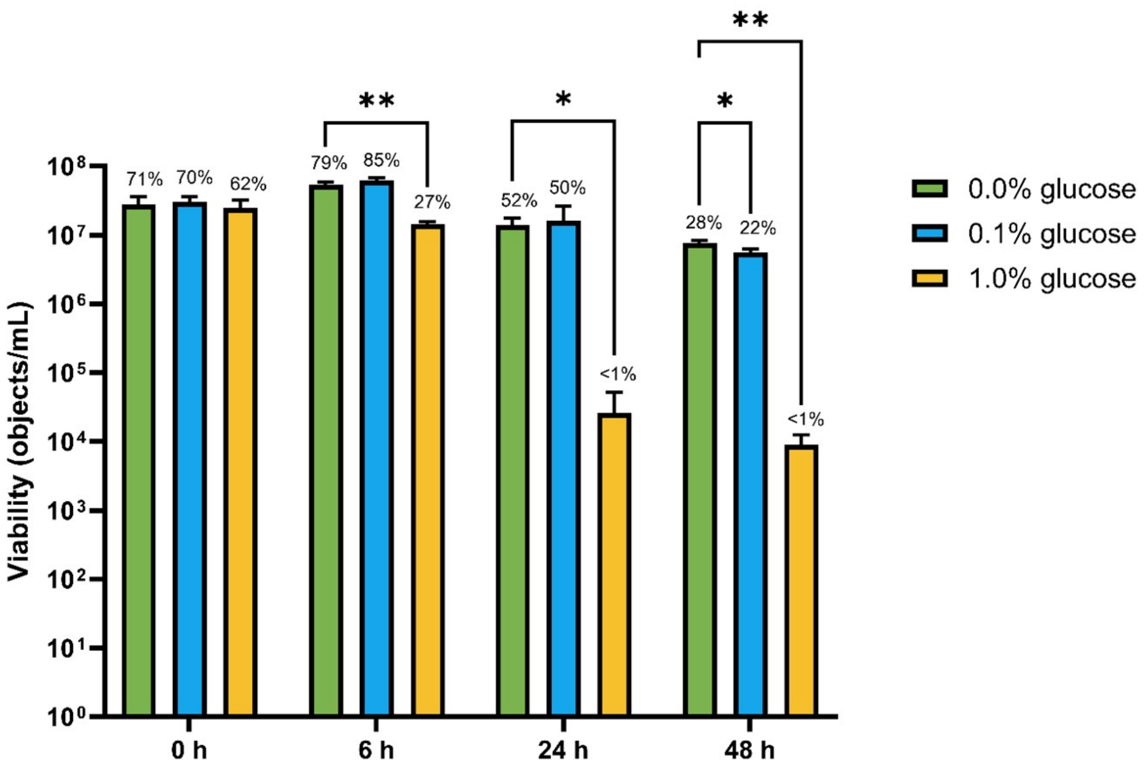

Supplementary Figure S2. The total viable bacterial cells (objects/mL) and percent viable cells of total cells (%) per treatment in high-throughput colon models treated with different concentrations of glucose (0, 0.1, 1.0%). *Viability was determined by flow cytometry, using an ImageStream^{X} Mk II (Cytek Biosciences) imaging flow cytometer. Results are shown as mean ± SD of three biological replicates from one donor. Statistical analysis employed Two-Way ANOVA with post-hoc pairwise comparisons between each glucose concentration and the control (0% glucose) per time point (* P < 0.05, ** P < 0.01).*

Supplement 3

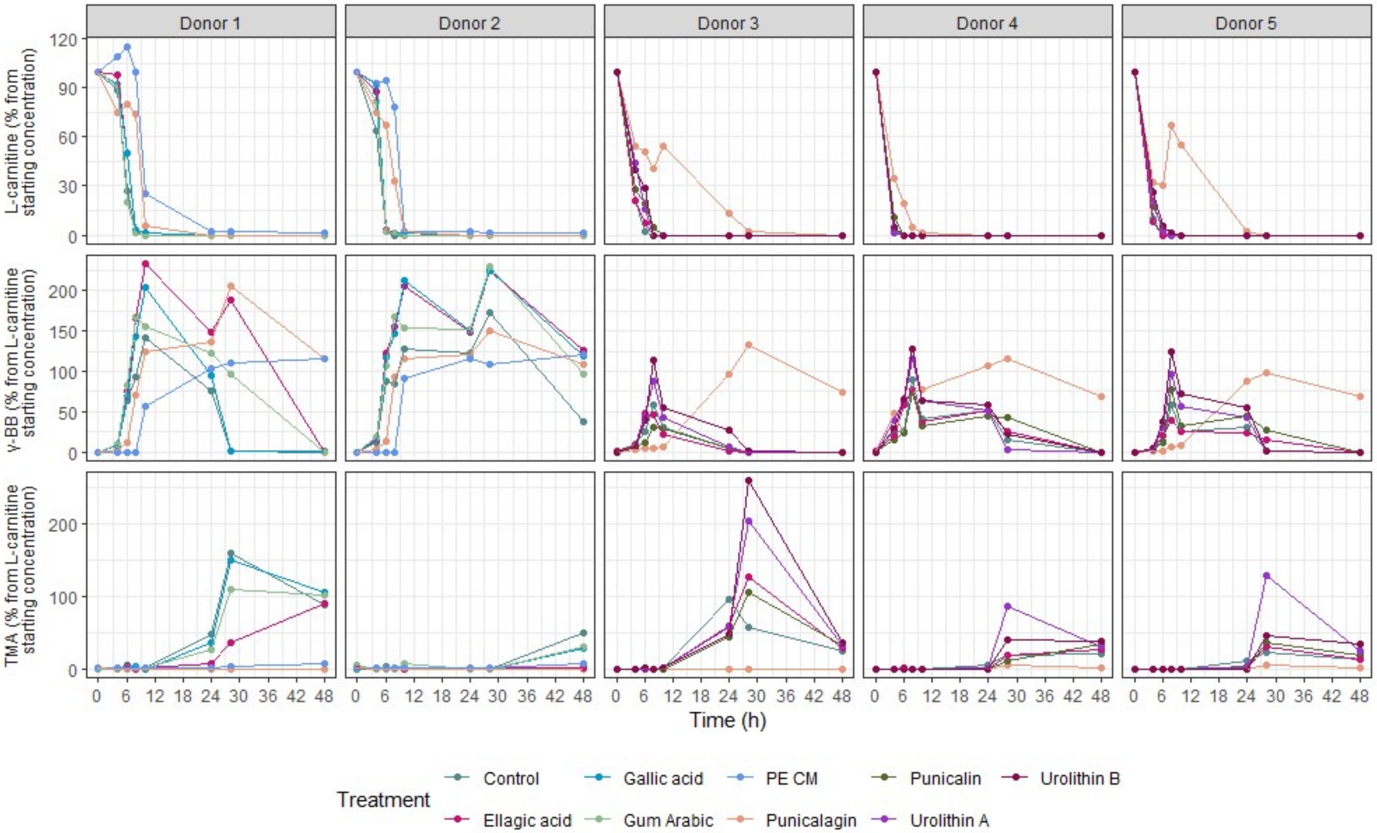

Supplementary Figure S3. Effects of selected polyphenols, urolithins, and gum Arabic at 2 mg/mL on **in vitro** ***L-carnitine metabolism, stratified by individual donors****. Average percentages of L-carnitine, γ-butyrobetaine (γ-BB), and trimethylamine (TMA) relative to initial L-carnitine concentration are displayed over 48 hours, with 3-4 replicates per donor. High-throughput* in vitro *colon models were inoculated with 1% faecal inoculum from a healthy donor, 2 mM L-carnitine, and the treatment. After collection, samples were directly stored at -80 °C until LC-MS/MS quantification using isotope-labelled internal standards. For comparison, the trajectory of pomegranate extract (22.8 mg/mL) in a pH-controlled* in vitro *batch colon model (PE CM) was included, as described in a previously published report* [1].

References

1. J. E. Haarhuis, P. Day-Walsh, E. Shehata, G. M. Savva, B. Peck, M. Philo and P. A. Kroon, A Pomegranate Polyphenol Extract Suppresses the Microbial Production of Proatherogenic Trimethylamine (TMA) in an In Vitro Human Colon Model, *Molecular Nutrition & Food Research*, 2025, **69**, e70166.
